# Return on investment of internet delivered exposure therapy for irritable bowel syndrome: a randomized controlled trial

**DOI:** 10.1186/s12876-021-01867-6

**Published:** 2021-07-13

**Authors:** Hugo Wallén, Perjohan Lindfors, Erik Andersson, Erik Hedman-Lagerlöf, Hugo Hesser, Nils Lindefors, Cecilia Svanborg, Brjánn Ljótsson

**Affiliations:** 1 Department of Clinical Neuroscience, Division of Psychology, Karolinska Institutet, Nobels väg 9, 171 65 Stockholm, Sweden; 2 School of Law, Psychology and Social Work, Center for Health and Medical Psychology, Örebro University, Örebro, Sweden; 3 Department of Behavioural Sciences and Learning, Linköping University, Linköping, Sweden; 4 Centre for Psychiatry Research, Department of Clinical Neuroscience, Karolinska Institutet, Stockholm, Sweden; 5 Stockholm Health Care Services, Region Stockholm, Karolinska University Hospital, 141 86 Stockholm, Sweden

**Keywords:** IBS, Cognitive behavior therapy, Cost-effectiveness analysis, Return on investment, Internet, Exposure

## Abstract

**Background:**

Irritable bowel syndrome (IBS) is a debilitating and costly disorder. Cognitive behavior therapy (CBT) is effective in the treatment of IBS, both when delivered over the internet and in face-to-face settings. CBT consists of different components and little is known about their relative importance. We have in an earlier study showed that inclusion of exposure in the CBT for IBS makes it even more effective. In the present study we wanted to evaluate the economic effects for society of inclusion vs exclusion of exposure in an internet delivered CBT for IBS.

**Methods:**

We used data from a previous study with 309 participants with IBS. Participants were randomized to internet delivered CBT with (ICBT) or without exposure (ICBT-WE). We compared direct and indirect costs at baseline, after treatment, and 6 months after treatment (primary endpoint; 6MFU). Data was also collected on symptom severity and time spent by therapists and participants. The relative Incremental Cost Effectiveness Ratio (ICER) was calculated for the two treatment conditions and the return on investment (ROI).

**Results:**

Results showed that ICBT cost $213.5 (20%) more than ICBT-WE per participant. However, ICBT was associated with larger reductions regarding both costs and symptoms than ICBT-WE at 6MFU. The ICER was − 301.69, meaning that for every point improvement on the Gastrointestinal Symptom Rating Scale—IBS version in ICBT, societal costs would be reduced with approximately $300. At a willingness to pay for a case of clinically significant improvement in IBS symptoms of $0, there was an 84% probability of cost-effectiveness. ROI analysis showed that for every $1 invested in ICBT rather than ICBT-WE, the return would be $5.64 six months after treatment. Analyses of post-treatment data showed a similar pattern although cost-savings were smaller.

**Conclusions:**

Including exposure in Cognitive Behavior Treatment for IBS is more cost-effective from a societal perspective than not including it, even though it may demand more therapist and patient time in the short term.

**Trial registration:**

This study is reported in accordance with the CONSORT statement for non-pharmacological trials [1]. Clinicaltrials.gov registration ID: NCT01529567 (14/02/2013).

## Background

Irritable bowel syndrome (IBS) affects one out of ten persons making it one of the most common chronic disorders in the world [2]. The IBS diagnosis is based on the Rome IV criteria [3], which state that core symptoms for diagnosis are abdominal pain in combination with diarrhea and/or constipation. More severely affected IBS patients also show behavioral and psychological symptoms, including avoidance behavior, social discomfort, rumination, hypersensitivity, stress, and depression [4]. IBS is not only a disabling disorder for the individual, but also constitutes a large economic burden for society [5]. Costs for medical examinations, treatments, drugs and sick leave have been equated with the economic consequences of the flu [6]. The estimated direct healthcare costs for IBS in the United States have been estimated to about $2 billion per year [7], while the combined direct and indirect costs were estimated to $30 billion per year [8]. A Finnish study from 2010 concluded that costs incurred by IBS amount to 5% of the national direct outpatient and medication costs [9].

If basic management of IBS does not lead to satisfactory results, psychological treatment can be an effective approach [10]. Cognitive behavior therapy (CBT) is the most researched treatment and the most effective in improving daily functioning [11]. Our research group has developed a CBT protocol for IBS that is based on the role of gastrointestinal symptom-specific anxiety (GSA) in the maintenance and exacerbation of symptom severity and disability in IBS [12]. GSA is a specific form of fear of and preoccupation with IBS-related stimuli or situations. Patients with high GSA avoid behaviors that could provoke symptoms (e.g. specific food) and situations in which the symptoms would be embarrassing (e.g. being far away from a toilet). High GSA has been shown to worsen symptom severity of IBS and to reduce quality of life [12–15] and treatments that target GSA tend to have large effects on IBS symptoms and quality of life [16].

Our GSA-directed CBT protocol includes four different treatment components; psychoeducation, mindfulness training, values-based behavior change, and systematic exposure to aversive IBS-related stimuli. The purpose of systematic exposure is to achieve long-term reduction of anxiety associated with these stimuli and consequently reduce the severity of symptoms and disability. The protocol has been evaluated in face-to-face format [17, 18] and as Internet-delivered CBT (ICBT) in a series of randomized controlled trials (RCTs) [19, 20]. The studies have consistently demonstrated large reductions in IBS symptom severity and disability. In the most recent RCT, we investigated the relative importance of the exposure component compared to the other components that are included in the treatment (i.e., psychoeducation, mindfulness training, and values-based behavioral change). In the study, 309 participants with IBS were randomized to treatment either with or without the specific exposure component [21]. The hypothesis was that exposure is necessary to reduce GSA [14] and thus that exposure specifically would have beneficial treatment effects. The study showed that inclusion of exposure had an incremental treatment effect [21, 22] and that the effects of exposure were mediated through reduced GSA (i.e., avoidance behavior and symptom-worry) [23].

Exposure therapy clearly makes CBT for IBS more efficacious, but does it also make the treatment more expensive? For most medical conditions, new and more efficient, but not always more cost-effective, pharmacotherapies are developed continuously [24]. Psychological treatments (particularly CBT) are cost-effective for many conditions [25, 26] and we have also found indication that the exposure-based ICBT for IBS is cost-effective compared to waitlist control [19, 27]. However, the relative cost-effectiveness of the exposure-based treatment compared to a waitlist does not necessarily mean that ICBT with exposure would be more cost-effective than ICBT without exposure, even though the latter is more effective. Exposure exercises are demanding for patients, possibly making the treatment more time consuming for both patients and therapists and hence more expensive. On the other hand, a more efficacious treatment that reduces both IBS symptoms and IBS-related avoidance behavior can potentially reduce societal costs. Improvement in IBS symptom could reduce the perceived need for healthcare and pharmacological treatment and increase work productivity. Avoidance behavior, such as using of over-the-counter medications and staying home from work because of symptoms, can also be cost driving.

The aim of the present study was to investigate the treatment costs of the two different treatment protocols (ICBT with exposure and ICBT without exposure; ICBT-WE) in relation to their effects on symptom severity and to their potential to save societal costs (i.e. costs for medical examinations, treatments, medications, and sick leave). The hypothesis was that exposure—by providing patients with better strategies to handle symptoms and/or by reducing the severity of symptoms—would reduce the need for health care and sick leave more than the same treatment without exposure and thereby make up for its potentially higher direct treatment costs. In addition to a traditional health economic analysis, we also analyzed whether an investment in ICBT would yield a larger return from a societal perspective than the same investment in ICBT-WE. For that purpose, we also used a corporate economic stand point and performed a “Return on Investment” analysis [28].

## Methods

### Design of the study

Participants treated with ICBT or ICBT-WE for IBS in our previous study [21] provided data regarding their symptoms, direct and indirect costs associated with their condition. We collected data before treatment, after treatment and 6 months after treatment (6MFU). The main aim of the original study was to investigate whether the exposure component protocol would yield an incremental treatment effect or not. The present study was designed as a prospective cost-effectiveness analysis with a societal perspective. By comparing the costs and gains for the two different treatment conditions with their treatment efficacy an incremental cost effectiveness ratio (ICER) could be calculated to demonstrate how much one unit of improvement would cost in one treatment condition relative to the other. The trial was pre-registered with clinicaltrials.gov (Identifier NCT01529567) (14/02/2013). All participants provided written informed consent. The study was approved by the regional ethical review board in Stockholm (Identifier 2011/1536-31/3).

### Participants and recruitment

Participants were recruited through self-referral and information about the study was spread through several channels, for example newspaper advertisements, an online discussion forum about IBS, and a web portal for internet-based treatment studies. Participants received no compensation for their participation in the treatment. Self-report assessments were collected using a secure online assessment system. Internet administration has been shown to be a reliable format for measures of psychiatric symptoms and quality of life [29].

The total sample comprised 309 adult participants with IBS, of whom 153 were randomized to ICBT and 156 to ICBT-WE. The mean age was 42.4 years (SD = 14.5). There were 246 women (79.5%) and the participants had been diagnosed with IBS 8.4 years ago on average (SD = 9.4, median 5.0). The study was conducted at the Internet Psychiatry Unit in Stockholm, Sweden, but recruitment was carried out nationwide. Inclusion criteria were (1) previous diagnosis of IBS given by a physician, (2) fulfilment of Rome III-criteria for IBS [30], (3) age 18 years or older. Participants were excluded from the study if they reported uninvestigated alarm symptoms (symptoms that have been shown to predict presence of more serious organic diseases and warrant further medical examination [31]) or severe psychiatric symptoms. A more detailed description of the recruitment procedure is available in the original paper [21].

### Treatments

The treatments were accessible through an online platform and lasted for 10 weeks. Using the internet to provide psychological treatment has been shown to be effective [32] and is well-accepted by healthcare providers [33]. Both treatment conditions were divided into successive steps that participants had to complete in order to gain access to the next. Each step contained educational material and exercises with rationales and instructions. The first three steps were the same in both conditions, starting with an explanation of how behaviors with the purpose of reducing symptoms may cause the participant to focus attention to symptoms and hence become hypervigilant towards bodily sensations. Brief mindfulness exercises were also taught in the first step. The second step described the psychological model for how anxiety and IBS might mutually reinforce each other. The third step gave tools for identifying life values and explained how to shift focus from a life governed by IBS symptoms to achieving personal goals and living a life in accordance with personal values. The ICBT condition also included a fourth step focusing on exposure to feared or avoided IBS-related situations. In the ICBT-WE condition the participants were encouraged to reach step 3 in three weeks and work with values-based behavior change until the last week. In the ICBT condition, participants were encouraged to reach step 4 in four weeks and work with values-based behavior change and exposure for the remainder of the treatment. Participants in ICBT were instructed and guided to systematically and repeatedly expose themselves to IBS-related situations and stimuli that elicited fear and distress. Participants in both arms were instructed to reduce avoidance and control behavior to become less affected by worry about symptom and symptom flare-ups. The purpose of the exposure exercises in the ICBT arm were facilitate learning that the bodily cues and situations that participants feared are tolerable and unlikely to lead to catastrophic consequences [34]. Both treatments were concluded with a text about relapse-prevention. More details about the treatment content in both arms are found in the original publication [21].

Throughout treatment, participants communicated with a therapist (licensed psychologists or students in the last semester of their master program in psychology—in the following referred to as “psychologists”) asynchronously through an email-like messaging system. Participants were encouraged to send at least one message per week to their treating psychologist. The psychologist answered messages within 2–3 days. The psychologists’ communication had mainly three purposes: (1) Correct misunderstandings or facilitate learning from the educational material; (2) coach the participant to increase or maintain work with the treatment; and (3) guide the participant through exercises prescribed by the treatment.

### Clinical outcome assessment

The outcome measures were assessed at three time points; pretreatment, post-treatment, and 6MFU. The primary outcome measure of the study was the Gastrointestinal Symptom Rating Scale—IBS version (GSRS-IBS; [35]), which has demonstrated good psychometric properties for the different symptoms that are assessed, with an internal consistency (Cronbach’s α) of 0.88 [36].

### Cost assessment

Cost data were collected with the Trimbos/iMTA questionnaire for Costs associated with Psychiatric illness (TIC-P; [37]). It is a self-report measure that covers three economic domains: direct medical costs, indirect medical costs and indirect non-medical costs. Direct medical costs refer to healthcare consumption, for example primary care visits or tertiary gastroenterological consultations. Indirect medical costs include costs associated with the clinical symptoms (i.e. pain) but not considered healthcare, for example attending self-help groups. Finally, indirect non-medical costs are related to loss of productivity, costs for sick leave or domestic productivity loss.

Costs of productivity loss, were calculated by multiplying the days the participant reported to have been at sick leave, had lower production at work or been unable to do domestic work with the mean daily income in Sweden obtained from “Statistics Sweden” [38]. The costs were initially assessed in Swedish krona (SEK) and converted into US$ using 2017 as the reference year, yielding a 8.719 SEK equivalent of 1 US$ [39]. Costs of healthcare services and medications were, when available, obtained from official healthcare tariff indexes for services offered within the publicly funded healthcare system.

The estimated treatment costs of ICBT and ICBT-WE were based on the time that participants and therapists spent on the treatment. Therapist time was measured by the treatment platform by logging the time therapist spent communication with the participants. Participant time was based on weekly self-reported number of hours that participant had spent reading the treatment material and performing treatment exercises. Because of administrative error, participant hours were not collected for the first week of treatment. The missing number of hours was imputed based on the individual mean number of hours during weeks 2–10. Cost for therapist time was based on tariffs for licensed psychologists ($381) while cost for participant time ($18) was based on the cost for domestic work. The costs used and how they were retrieved are presented in Table 1.

**Table 1:** Cost tariffs for the most common types of health services utilized by the participants

Type of cost	Unit	Price^a^
General practitioner	Consultation	381^b^
Occupational physician	Consultation	381^b^
Nurse	Consultation	285^b^
Social worker	Consultation	261^b^
Physiotherapist	Consultation	127^b^
Psychologist or psychotherapist at private clinic	Session	126
Psychologist or psychotherapist at a primary care unit	Session	381^b^
Psychologist or psychotherapist at a psychiatry unit	Session	381^b^
Psychiatrist	Consultation	465^b^
Medical specialist	Consultation	357^b^
Communal home care (including cleaning and babysitting)	Hour	47^c^
Alternative medicine (examples include homeopaths and acupuncturists)	Consultation	104^d^
Self-help group (e.g., support group within a patient association)	Hour	18^e^
Searching medical information on the internet or taking part in online patient networks	Hour	18^e^
Informal care (relatives and friends)	Hour	18^e^
Sick leave	Hour	22^f^
Presenteeism at work	Hour	22g
Inability to do household work	Hour	18^e^
Presenteeism in household work	Hour	18 g

^a^Costs in US dollars for 2017, Power Purchasing Parity (PPP) derived from OECD[39]

^b^Stockholm County Council tariffs 2017[40]

^c^National tariff from Swedish Association of Local Authorities and Regions adjusted to 2017 with consumer price index (CPI) [41]

^d^A mean from 5 different google searches with 8 different treatments. Adjusted with CPI for 2017

^e^Cost used from a Dutch study, but assumed to be equal in Sweden [42]

^f^Average monthly Salary 2017, from Statistics Sweden, adjusted with gender differences in the sample [38]

^g^Tariff multiplied with percentage of total time, as reported by participants

### Data analysis

We used SPSS v. 22.0 (IBM) and R version 3.5.3 [43], and the R packages nlme version 3.1–137 [44] and Influence.ME version 0.9–9 [45] to conduct the statistical analyses. All estimates, unless otherwise noted, were based on linear mixed models analysis with random intercept to account for dependence between assessment points. The mixed models included all available observations at the three timepoints and thus constituted intent-to-treat analyses. To allow for different development over time from pre- to post-treatment and from post-treatment to 6MFU, the models included two time variables, one describing the first time period (T1) and one describing the second time period (T2; i.e., piecewise regression analysis). The analyses also included the group variable and its interaction effect with the two-time variables T1 and T2 to estimate the differential development over time for the two groups during these distinct phases in the trial.

The recall period of the TIC-P is one month, meaning that the costs obtained at post-treatment were incurred during the last month of treatment. These post-treatment costs could potentially be overestimated because possible effects of treatment on healthcare utilization would not have had full effect. However, post-treatment costs could also be underestimated because participants would potentially have consumed more healthcare if they had not participated in the study. Because of these potentially confounding factors in the post-treatment costs, we therefore based the main results on the estimated changes in costs and symptom outcomes from pre-treatment to 6MFU. This change was estimated by summing the interaction effects T1*group and T2*group from the piecewise mixed models. Results based on post-treatment data are briefly reported and available from the authors upon request.

### Cost-effectiveness analyses

The incremental cost-effectiveness ratio analyses were based on estimated changes in symptom severity and costs in the two groups. All societal costs were extrapolated to 6 months (i.e. multiplied with six) and the treatment costs (therapist and participant time) were added to the costs after treatment (i.e., 6MFU and post-treatment costs). The ICER was calculated as the difference between the groups in estimated costs change from before and after treatment divided by the difference between the groups in estimated change in symptom severity from before to after treatment. We used the equation (Δ^C1^–Δ^C2^)/(Δ^E1^–Δ^E2^) where Δ^C1^–Δ^C2^ is the net difference in cost change and Δ^E1^–Δ^E2^ is the net difference in effectiveness (i.e., symptom improvement) of the two treatments.

The analyses were repeated in 5000 bootstrap replications to obtain confidence intervals of the ICER estimate and to visualize the spread of the ICERs in a graph. We also calculated the willingness to pay (WTP; the probability that treatment is cost effective compared to control group if one is willing to pay X dollars per unit of symptom improvement) and plotted different WTP cut-offs in the ICER graphs.

Symptom improvement was calculated in two different ways. First based on individual change in GSRS-IBS total score and then based on achieving clinically significant improvement. The criterion for clinically significant improvement was a reduction in GSRS-IBS score of at least 30% [46, 47]. Because the percent improvement calculation requires the post-treatment status to be known, these analyses were based on complete cases rather than all available data. Based on data in the second analyses, we also calculated the number needed to treat (NNT), that is, how many participants would need receive exposure therapy to achieve one more case of clinically significant improvement compared to the control group. The NNT calculation was made by inversing the absolute risk reduction, that is, dividing 1 by the difference between the groups in proportion to who achieved clinically significant improvement.

### Return on investment

We also calculated the return on investment ratio (ROI) of the ICBT treatment relative to the ICBT-WE treatment. The concept of ROI is often used to calculate whether an investment will be worth its costs by producing a larger net value than else would have been the case [48, 49]. ROI is calculated by subtracting the cost of the investment from the total gains and then dividing the result with the cost of the investment. The results show how much every unit of investment yields in higher value of service. In the case of health care, the value of service is analogous to saved costs (i.e., future costs that the society will not have to pay because the participant is offered a more efficacious treatment). To get the ROI in the ICBT treatment relative to the ICBT-WE treatment we used the formula (Gain-Investment)/Investment) = ((C^1pre^) − (C^1FU^) − (C^2pre^) − (C^2FU^)) − (I^2^ − I^1^)/(I^2^ − I^1^). In the formula, C^1^ represent (societal) costs in the ICBT group, C^2^ represent (societal) costs in the ICBT-WE group, I^1^ represent treatment costs (investment) in the ICBT group and I^2^ represent treatment costs (investment) in the ICBT-WE group.

### Sensitivity analyses

The costs used in our calculations were obtained from Stockholm County Council [40]. The cost data are accurate but are based on activity rather than the medical specialty of the treating physician. We set the cost for a general practitioner (GP) visit to the cost of a bowel-related disorder visit (381 USD), which was higher than the costs for a visit regarding an unspecified disorder to a medical specialist (357 USD). However, GP visits are generally regarded to cost less than costs for visits to medical specialists and thus our calculations would not apply if the actual GP visits were made for other reasons than bowel related disorders. We therefore investigated the robustness of the results by rerunning the main analysis with GP visit cost set to the cost that Stockholm City Council charges other healthcare regions for a GP visit, i.e., 206 USD [50].

### Outlier analyses

Cost data typically show large variations and statistical analyses of these data are sensitive to outliers. We therefore performed sensitivity analyses to investigate the possible impact of cost outliers in the data. We used the Influence.ME package to identify influential data in the piecewise regression of cost-change in the two groups over the study period. Potential outliers were identified on both observation-level and at the individual level using visual inspection of Cook’s distance plots. The main outcome analysis (i.e., cost-effectiveness of one-point GSRS-IBS improvement at 6MFU) was then rerun with 5000 bootstrap replications, with one of the potential outlier observations removed per rerun. The influence of each removed outlying observation was then determined based on the shift of the cost-effectiveness quadrants distributions (see explanation below) compared to the original analysis.

## Results

### Attrition and IBS outcome

The proportion of participants that completed all steps of the treatments were 55% in the ICBT group and 60% in the ICBT-WE group [22]. All the 309 participants completed assessment at baseline and 285 completed post-treatment assessment (94.5%) and 259 completed assessment at FU (87.1%) (see Table 2). Participants who did not complete full assessment at post-treatment and FU were given the possibility to complete a reduced assessment battery that included the GSRS-IBS but not the TIC-P. Thus, more data were available for the GSRS-IBS than the TIC-P.

**Table 2:** GSRS-IBS scores for the study groups at each assessment point

	Pre-treatment		Post-treatment		6MFU	
	ICBT	ICBT-WE	ICBT	ICBT-WE	ICBT	ICBT-WE
N	153	156	146	146	134	135
Mean	46.13	47.52	32.82	38.22	32.21	37.30
SD	10.19	10.97	11.39	14.49	12.34	13.37
Missing (%)	0 (0)	0 (0)	7 (4.6)	10 (6.5)	19 (12.4)	21 (13.7)

GSRS-IBS: Gastrointestinal Symptom Rating Scale—IBS version. 6MFU: six months after treatment. ICBT: Internet delivered Cognitive Behavior Therapy including exposure. ICBT-WE: Internet delivered Cognitive Behavior Therapy without exposure

We investigated the data missingness pattern by comparing baseline data between patients who did and did not have missing cost data at 6MFU, separately for each group. We found that patients with missing 6MFU costs in the ICBT-WE group had significantly higher costs at baseline, missing = 2405.63 (SD = 2486.86), not missing = 1465.12 (SD = 1570.05), t(154) = − 2.55, *p* = 0.01, and higher GSRS-IBS scores, missing = 51.50 (SD = 12.86), not missing = 46.65 (SD = 10.36), t(154) = − 2.14, *p* = 0.03, than ICBT-WE patients who did not have missing 6MFU data. We saw a similar but non-significant pattern in the ICBT group, costs: 2090.04 (SD = 2826.32) vs. 1366.60 (SD = 1555.39), t(151) = − 1.76, *p* = 0.08; GSRS-IBS: 48.47 (SD = 12.78) vs. 45.73 (SD = 9.70), t(151) = − 1.16, *p* value = 0.25). Thus, missing 6MFU costs were associated with higher baseline costs and symptom severity in the ICBT-WE group but not the ICBT groups. We therefore assumed that analyses without imputing missing 6MFU data would potentially give more conservative estimates of cost-effectiveness than if the missing 6MFU data was imputed and relied on full information mixed models with restricted maximum likelihood estimation to provide the estimates of cost-effectiveness [51].

## Observed costs

The cost data collected with TIC- P is shown in Table 3. In the ICBT group all costs except for unemployment were reduced from pre-treatment to 6MFU. In the ICBT-WE group all costs were reduced. The treatment costs were higher in the ICBT group than in the ICBT-WE group due to more time spent in treatment from both therapists and participants. The mean time spent by therapists in ICBT vs ICBT-WE were 1.65 and 1.38 h, respectively, t(307) = 0.27, *p* = 0.061, 95% CI (− 0.01, 0.54) and the mean time spent by participants in ICBT vs ICBT-WE were 36.11 and 29.89 h respectively, t(307) = 6.22, *p* = 0.158, 95% CI (− 2.42, 14.86).

**Table 3:** Observed costs by type of expenditure for the study groups at each assessment point

	Pre (n)				Post (n)				6MFU (n)			
	ICBT (153)		ICBT-WE (156)		ICBT (141)		ICBT-WE (144)			ICBT (131)	ICBT-WE (128)	
Cost	Mean	SD	Mean	SD	Mean	SD	Mean	SD	Mean	SD	Mean	SD
Direct Medical	471.9	718.6	486.8	662.0	325.1	570.2	405.2	655.0	311.5	530.6	462.6	890.2
Healthcare visits	459.2	716.0	472.9	656.0	310.3	567.5	390.6	649.4	299.4	526.4	452.7	884.1
Medication	12.7	24.2	13.9	20.9	14.7	33.3	14.6	22.5	12.1	25.1	9.9	16.3
Direct non-medical costs	96.6	439.4	66.7	133.2	38.4	117.2	72.8	227.2	49.7	114.7	55.4	135.7
Indirect non-medical costs	902.1	1321.3	1080.4	1463.5	791.1	1366.2	986.1	1445.1	706.0	1269.7	879.6	1343.2
Unemployment	343.2	1084.9	480.8	1257.9	478.8	1256.0	546.9	1328.3	429.4	1198.8	351.6	1097.5
Sick leave	245.8	777.1	299.1	767.7	157.9	587.4	197.6	567.6	138.9	554.0	262.3	690.8
Work cutback	187.4	304.5	182.4	288.0	88.1	192.6	150.4	309.4	71.5	162.9	180.5	469.5
Domestic	125.7	223.6	118.2	228.7	66.3	141.4	91.1	249.8	66.2	150.3	85.1	186.3
Net total costs	*1470.6*	*1799.3*	*1633.9*	*1796.7*	*1154.5*	*1651.2*	*1464.1*	*1870.1*	*1067.2*	*1561.8*	*1397.6*	*1851.1*
Therapist time cost					627.6	521.1	526.2	422.2				
Participants time cost					650.3	559.0	538.3	806.6				
Total Intervention costs					*1277.9*	*937.0*	*1064.4*	*980.5*				

ICBT: Internet delivered Cognitive Behavior Therapy including exposure. ICBT-WE: Internet delivered Cognitive Behavior Therapy without exposure

All costs in USD

## Cost-effectiveness analyses

Table 4 shows the estimates obtained in the bootstrapped piecewise mixed models regression analyses on the GSRS-IBS and cost data. We used the estimated GSRS-IBS scores and costs derived from our mixed models analyses to calculate the ICER by dividing the relative change in costs from pre to 6MFU (ICBT − 627.95; ICBT-WE 567.73) with the relative change in GSRS-IBS score from pre to 6MFU (ICBT 13.52; ICBT-WE 9.56). The resulting ICER − 1195.68/3.96 = − 301.69 indicates that reducing GSRS-IBS with one point in the ICBT group compared to the ICBT-WE group incurred a societal gain of $302.

**Table 4:** Estimates from bootstrapped mixed models analyses of GSRS-IBS and costs

Estimate	B	s.e	95% CI	B	s.e	95% CI
Intercept	47.52	0.98	[45.82, 49.23]	9804	867	[8181, 11557]
Group	− 1.39	1.39	[− 3.76, 0.92]	− 980	1233	[− 3451, 1448]
T1	− 9.09	0.84	[− 10.74, − 7.54]	66	754	[− 1085, 1236]
T1*Group	− 3.87	1.19	[− 6.12, − 1.64]	− 467	1072	[− 2340, 1411]
T2	− 0.47	0.87	[− 1.96, 1.07]	502	793	[− 1107, 2131]
T2*Group	− 0.09	1.23	[− 2.31, 2.13]	− 729	1118	[− 2979, 1473]
T1*Group + T2*Group	− 3.96	1.23	[− 6.62, − 1.32]	− 1196	1110	[− 3550, 1256]

95% CI: Bootstrapped 95% confidence interval. GSRS-IBS: Gastrointestinal Symptom Rating Scale—IBS version

The estimates are from bootstrapped piecewise mixed models regression analyses of GSRS-IBS scores and total costs, including intervention costs. The T1 and T2 variables denote the time from pre-treatment to post-treatment and from post-treatment to 6-month follow-up, respectively. The T1 and T2 interaction effects with the Group variable denote the differential developments over time between the study groups during these two time periods. The summed interaction effect is the cumulative difference in change between pre-treatment to 6-month follow-up

**Fig. 1:**
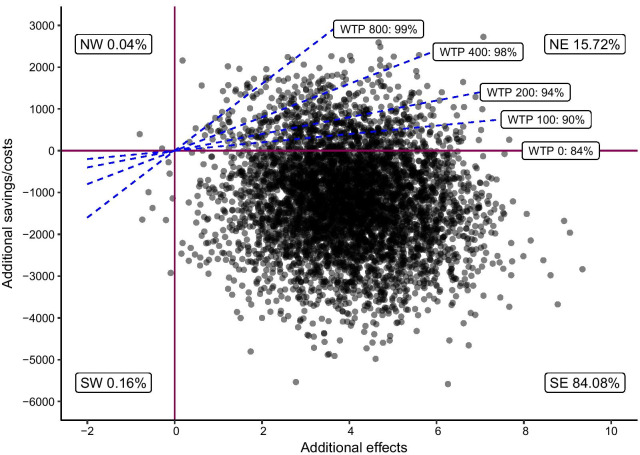
Cost-effectiveness plane comparing ICBT to ICBT-WE

The bootstrapped distributions of relative cost and symptom changes with 5000 replications are shown in Fig. 1. On the x-axis the relative effectiveness of the ICBT to ICBT-WE is shown and, on the y-axis, the relative savings for the ICBT compared to ICBT-WE is shown.

ICERs located in the northwest quadrant indicate that ICBT is less effective than ICBT-WE and is associated with higher societal costs whereas ICERs in the southeast quadrant indicate that ICBT is more effective and associated with lower societal costs. As can be seen in Fig. 1, about 84% of the bootstrapped simulations were located in the southeast quadrant, almost 16% were located in the northeast quadrant, and 0.2% were located in the western quadrants. The WTP scenario is the probability that the ICBT treatment will be cost-effective relative to ICBT-WE if society is prepared to spend a given amount per point of reduction on the GSRS-IBS. Figure 1 shows that at a WTP of $0, the intervention had an 84% probability of being cost-effective and 99% probability of cost-effectiveness was achieved at WTP $800.

The X-axis shows relative effects between the two treatments. A higher value indicates treatment effectiveness in favor of ICBT. The Y-axis shows relative costs between treatments with a lower value indicating cost savings. Willingness to pay (WTP) is shown in dollars and the percentage refers to the probability for cost-effectiveness given the willingness to spend that amount of money per point reduced GSRS-IBS score.

## Clinically significant improvement

**Fig. 2:**
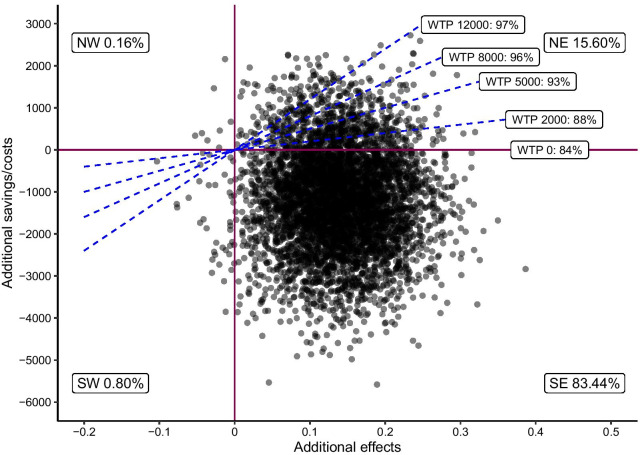
Cost-effectiveness plane comparing ICBT to ICBT-WE for clinical improvement

The proportion of participants who showed clinically significant improvement from pre to 6MFU was 52% in the ICBT-WE group and 66% in the ICBT group, a difference of 14 percentage points. The cost difference was $− 1195.68, resulting in an estimated ICER of − 8651.94. Thus, society would reduce its costs by $8652 for every patient who was clinically significant improved from the ICBT treatment, relative to ICBT-WE. As can be seen in Fig. 2, there was an 84% probability that a participant would show clinically significant improvement in the ICBT-group compared to ICBT-WE at a WTP of $0. If WTP was increased to $12,000, there was a 97% probability of cost-effectiveness.

The X-axis shows relative effects between the two treatments. A higher value indicates treatment effectiveness in favor of ICBT. The Y-axis shows relative costs between treatments with a lower value indicating cost savings. Willingness to pay (WTP) is shown in dollars and the percentage refers to the probability for cost-effectiveness given the willingness to spend that amount of money per patient that gets at least a 30% symptom reduction measured with GSRS-IBS.

## Numbers needed to treat

The percentage of participants that demonstrated clinically significant improvement (30% reduction or more on the GSRS-IBS) was 52% in the ICBT-WE group and 66% in the ICBT-group, yielding a NNT of 7.14.

## Return on investment

The ICBT group reduced their societal costs from pre to 6MFU with $2020.1 (over 6 months, excluding costs for treatment) and the ICBT-WE group reduced their costs with $602.6 over the same period. From a societal perspective, the gains of treating the participants with ICBT instead of ICBT-WE, were therefore $1417.5. The treatment costs based on therapist and participant time in the ICBT and ICBT-WE groups were $1278 and $1064 respectively with a slightly higher cost in the ICBT group due to more time spent in treatment. The extra intervention costs (investments) in the ICBT treatment were thus $214. The resulting return on investment in the ICBT group compared to the ICBT-WE group was 5.64, indicating that for every $1 spent on ICBT rather than ICBT-WE, society would gain $5.64 in reduced direct and indirect medical and health care costs. We also generated 5000 bootstrapped replications of the ROI calculation and found that 77% of the bootstrapped ROIs were above 1, which could be interpreted as a 77% probability that the exposure treatment would generate a positive return on investment.

## Results at post treatment

Estimated results at the post-treatment assessment showed a similar pattern of results but with smaller cost savings compared to 6MFU. The reduction in GSRS-IBS score in the ICBT and ICBT-WE groups were 12.97 and 9.09, respectively, and the cost changes were − 400.79 and 66.13, respectively. The resulting ICER was − 120.50, indicating that each incremental point improvement on the IBS-GSRS in ICBT relative ICBT-WE was associated with a societal cost reduction of $120. As for clinically significant improvement, 44% were improved in the ICBT-WE group and 66% in the ICBT group, resulting in an ICER of − 2065.79. The return on investment in the ICBT group relative to the ICBT-WE group at post was 2.31 and there was a 59.3% probability of ROI ≥ 1.

## Sensitivity analyses

The main analysis, cost-effectiveness at FU6, was rerun with costs for GP visits reduced to 206 USD (compared to 381 USD in the original analysis). The relative cost change was reduced to − 962.53 (compared to − 1195.68 in the original analysis) resulting in an ICER of − 242.86 (− 301.69 in the original analysis). On the cost-effectiveness plane, 79.88% of the simulated ICERs were located in the south-east quadrant (83.44% in the original analysis). Thus, the sensitivity analysis indicated that the cost for GP visits was not highly influential on the main analysis.

## Outlier analyses

We identified 7 possible outlier observations of costs from 4 individuals, 2 in the ICBT group and 3 in the ICBT-WE group. We also identified 8 individuals (5 in the ICBT group and 3 in the ICBT-WE group), whose overall contribution to the mixed models analysis of costs indicated that they were potential outliers. The main analysis of cost-effectiveness at FU6 was rerun with each of these observations and individuals removed. Because we only identified potential cost-data outliers, the quadrant shift occurred mainly between the south and north quadrants. The largest shift occurred when removing one individual’s observations in the ICBT-WE group which resulted in 76.80% of the simulations being in the south-east quadrant and 23.10% in the north-east quadrant (compared to 84.08% and 15.72% in the original analyses, respectively). Thus, although the largest shift because of one individual was about 7 percentage points in favor of ICBT-WE, the majority of the simulations with outliers removed were still located in the south-east quadrant, indicating robustness of the results.

## Discussion

We have previously demonstrated that internet-delivered CBT for IBS is more efficacious in terms of symptom reduction if an exposure component is included in the treatment. However, it was not known if including exposure leads to a more cost-effective intervention. The results from the present study showed that there was a 84% probability that ICBT was more cost effective than ICBT-WE at willingness to pay of $0, both when the calculations were based on reduction of one point on the GSRS-IBS and when based on a clinically significant reduction of the patients symptom burden (i.e. at least 30% reduction on GSRS-IBS). The relative return on investment for ICBT vs ICBT-WE was $5.64 indicating that exposure-based treatment is a sound investment for society despite its somewhat higher initial cost. Importantly, the results are based on six-month follow-up time. If the treatment effects and relative cost-savings were maintained after these six months, both the ICER and the ROI would grow as the treatment costs are amortized over a longer time. Overall, the results suggest that ICBT can be a highly cost-effective treatment compared to ICBT-WE in the treatment of IBS.

Because cost data often show non-normal distributions and each cost is associated with a fair amount of uncertainty, it is important to do sensitivity analyses in cost-effectiveness evaluations [52]. In the present trial, neither the sensitivity analyses that assumed lower costs for GP visits nor the outlier analyses showed any substantial effects on the primary analysis of cost-effectiveness at 6MFU. Thus, both these analyses indicated that the main results are robust.

Our results align with other studies in the field. Donker et al. (2016) showed that therapist guided ICBT for a range of psychiatric conditions is cost-effective compared to wait list, TAU, group treatment, attention control, telephone counseling, and unguided Internet CBT. Our findings add to the body of knowledge by showing that ICBT for IBS is more cost-effective if it includes systematic exposure to feared stimuli.

We regard the findings of this study to be of high clinical relevance. In recent years, there has been some debate regarding the drawbacks of exposure in psychological treatment [53, 54]. A critique that has been raised is that exposure may be perceived as negative by patients and mental health professionals and associated with dropout and feelings of stress [55]. However, few adverse events were reported in the original study and there was no difference between the groups in the number or severity of adverse events [21]. The present analysis further showed that, from a societal perspective, the increased costs associated with exposure are offset by its gains. Previous studies have concluded that societal costs incurred by IBS are not only dependent on the severity of IBS symptoms [56]. Abnormal illness behavior (i.e., how a patient perceives, evaluates and act on symptoms, similar to GSA) also plays an important role in health care consumption patterns in IBS [56] and studies on patients with chronic pain show that symptom fear and avoidance behavior is an important predictor of health care consumption [57]. It is therefore probable that exposure treatment leads to cost reduction through two distinct pathways, partly through its effect on symptom severity and partly through reduced gastrointestinal symptom-specific anxiety. Excessive healthcare seeking may be seen as an example of excessive control behavior, influenced by fear of or preoccupation with IBS symptoms. An important concept in the exposure treatment is to refrain from acting in response to symptoms, which may have reduced healthcare seeking.

Key strengths of our study are the large number of participants and the relatively small amount of missing data. The fact that the cost-effectiveness was more pronounced at 6MFU than at post-treatment indicates positive long-term effects and adds further validity to our conclusions.

A limitation in our study is that the societal costs undoubtedly included healthcare use and productivity losses that were not related to IBS. This increases cost variances and decreases the precision of the cost-effectiveness analysis, but nevertheless the results supported our hypotheses. It should also be mentioned as a limitation of the study that societal costs for health care may vary between countries, which limits the generalizability of the cost estimates to other countries. Another limitation is that not all patients completed the treatment and treatment completion was not taken into account in the present study. A secondary analysis of the data in the present study [22] suggested that the symptom improvements were substantially higher among the subgroup of participants who actually received the exposure component in ICBT. Thus, we may have similarly underestimated the effect of exposure on societal cost changes by including participants who did not complete the ICBT treatment. It may be noted that we did not include any costs for the development of the ICBT treatment manual, training of therapists, or the development and hosting of the digital platform that was used to deliver the treatments. However, while these costs may be of importance to health care providers when deciding on implementing internet delivered psychological treatment, therapist training and manual development are one-time costs and digital platform costs can often be shared between several healthcare providers and amortized over many patient groups that benefit from receiving treatment over the platform. Finally, the study was primarily designed to evaluate the efficacy in terms of symptom improvement of using exposure therapy for IBS. The power calculation was therefore not based on expected differences in and variances of cost data. Cost-effectiveness evaluations are often underpowered [58] and the estimates in the present study are associated with large uncertainty. Nevertheless, in the context of psychological intervention studies, the sample size was relatively large, and the proportion of missing data was small, lending credibility to overall results.

In conclusion, our findings in this study show that in addition to being an effective treatment for IBS symptoms, exposure based ICBT for IBS is a cost-effective treatment that has the potential to save societal costs. This knowledge is of importance when deciding on what interventions healthcare providers should offer for IBS.

## Data Availability

The datasets used and analyzed during the current study are available from the corresponding author on reasonable request.

## Abbreviations

6MFU: Six months follow-up
CBT: Cognitive behavior therapy
GSA: Gastrointestinal specific anxiety
GSRS: Gastrointestinal symptom rating scale
IBS: Irritable bowel syndrome
IBS-C: Irritable bowel syndrome, constipation predominant
IBS-D: Irritable bowel syndrome, diarrhea predominant
ICBT: Internet delivered cognitive behavior therapy
ICBT-WE: Internet delivered cognitive behavior therapy without exposure
ICER: Incremental cost-effectiveness ratio
NNT: Numbers needed to treat
OECD: Organization for economic co-operation and development
PPP: Power purchasing parity
RCT: Randomized controlled trial
ROI: Return on investment
SEK: Swedish krona
TAU: Treatment as usual
TIC-P: Trimbos/iMTA questionnaire for costs associated with psychiatric illness
USD: United States dollar
WTP: Willingness to pay
